# Probucol-Induced α-Tocopherol Deficiency Protects Mice against Malaria Infection

**DOI:** 10.1371/journal.pone.0136014

**Published:** 2015-08-21

**Authors:** Maria Shirely Herbas, Mototada Shichiri, Noriko Ishida, Aiko Kume, Yoshihisa Hagihara, Yasukazu Yoshida, Hiroshi Suzuki

**Affiliations:** 1 Research Unit for Functional Genomics, National Research Center for Protozoan Diseases, Obihiro University of Agriculture and Veterinary Medicine, Hokkaido, Japan; 2 Health Research Institute, National Institute of Advanced Industrial Science and Technology (AIST), Osaka, Japan; 3 Department of Basic Veterinary Science, The United Graduate School of Veterinary Sciences, Gifu University, Gifu, Japan; Ehime University, JAPAN

## Abstract

The emergence of malaria pathogens having resistance against antimalarials implies the necessity for the development of new drugs. Recently, we have demonstrated a resistance against malaria infection of α-tocopherol transfer protein knockout mice showing undetectable plasma levels of α-tocopherol, a lipid-soluble antioxidant. However, dietary restriction induced α-tocopherol deficiency is difficult to be applied as a clinical antimalarial therapy. Here, we report on a new strategy to potentially treat malaria by using probucol, a drug that can reduce the plasma α-tocopherol concentration. Probucol pre-treatment for 2 weeks and treatment throughout the infection rescued from death of mice infected with *Plasmodium yoelii* XL-17 or *P*. *berghei* ANKA. In addition, survival was extended when the treatment started immediately after parasite inoculation. The ratio of lipid peroxidation products to parent lipids increased in plasma after 2 weeks treatment of probucol. This indicates that the protective effect of probucol might be mediated by the oxidative stressful environment induced by α-tocopherol deficiency. Probucol in combination with dihydroartemisin suppressed the proliferation of *P*. *yoelii* XL-17. These results indicated that probucol might be a candidate for a drug against malaria infection by inducing α-tocopherol deficiency without dietary α-tocopherol restriction.

## Introduction

In 2013, the World Health Organization (WHO) estimated that there were 198 million cases of malaria resulting in 584,000 deaths [1]. Although chloroquine (CQ) represented the first-line drug for malaria treatment [2,3], emergence of CQ-resistant *Plasmodium* strains has made malaria treatment difficult, especially in endemic areas [4–6]. Currently, the treatment of malaria relies on artemisin-combined therapies [7,8]; however, the emergence of a resistant strain was reported [9–11]. Thus, the rapid appearance of resistant strains against antimalarial drugs calls for a rethinking of the current strategies for the treatment of this infectious disease in endemic areas.

It is well acknowledged that nutrition plays an important role in modulating morbidity and mortality of malaria infection [12]. For example, it has been reported that a particular dietary pattern of populations living in malaria-endemic areas provides a form of diet-mediated antimalarial prophylaxis that maximizes iron-mediated free radical production in infected erythrocytes [13]. African pastoral populations, which are heavy consumers of milk, appear to manifest a different adaptive pattern against malaria involving low intake of para-aminobenzoic acid, vitamin E, and iron compared with other groups [13]. Thus, dietary adaptation of traditional cuisines increases the oxidative stress and inhibits parasite proliferation [13]. The sensitivity of *Plasmodium* to oxidative stress has been widely addressed [14–16]. Remarkably, *Plasmodium* does not possess essential anti-oxidant enzymes such as catalase and a classical glutathione peroxidase [17,18], even though they are equipped with thioredoxin, peroxiredoxin and glutathione systems that protect them from oxidative stress [17–20].

Recently, we have reported that α-tocopherol transfer protein knockout (α-ttp^Δ^) mice showing undetectable plasma concentrations of α-tocopherol, the most biologically active form of vitamin E, were resistant against malaria and cerebral malaria [21]. This resistance was attributed to the parasite DNA damage derived from the high oxidative stress due to α-tocopherol deficiency [22]. We have also demonstrated that this protective effect can be reversed by feeding α-ttp^Δ^ mice with α-tocopherol-supplemented diets [21,22].

However, it is difficult to induce α-tocopherol deficiency by dietary control, because most foods such as cereal grains, beans and vegetable oils, contain significant amounts of α-tocopherol [23]. For this reason, it was believed that α-tocopherol deficiency is impossible to apply for clinical malarial therapy. However, we consider that clinical application of α-tocopherol deficiency would be possible if a drug that could induce α-tocopherol deficiency would be discovered.

Probucol, 4,4′-[(1-methylehylidene)bis(thio)]bis[2,6-bis(1,1-dimethylethyl) phenol], is a drug used for the treatment of hyperlipidemias [24] because it inactivates the adenosine triphosphate-binding cassette transporter A1-mediated cholesterol efflux [25,26]. Interestingly, it has been reported that the fractional decrease of the plasma concentrations of α-tocopherol in hypercholesterolemic patients were 14% after three years of treatment using 0.5 g of probucol twice a day [27]. Moreover, the plasma concentrations of α-tocopherol were reduced to 10% by addition of 1% w/w probucol to the diet in a mouse model [28].

Thus, we examined whether probucol has a protective effect against murine malaria.

## Materials and Methods

### Ethics Statement

All protocols were approved by a committee for the Animal Care and Use of Obihiro University of Agriculture and Veterinary Medicine (Permit Number: 25–106) and the Committee for the Experiments involving Animals of the National Institute of Advanced Industrial Science and Technology (Permit Number: 2013–026).

### Mice

Male and female C57BL/6J mice were purchased from CLEA, Japan (Tokyo, Japan), housed in polycarbonate cages, and maintained as specific pathogen-free animals in light-controlled (lights on from 5:00 to 19:00) and air-conditioned rooms at the National Research Center for Protozoan Diseases. The temperature and the humidity of the animal rooms were maintained at 24°C ± 1°C and 50% ± 10%, respectively. Mice were given tap water and standard diet (CE-2, CLEA Japan; Tokyo, Japan) *ad libitum*. The number of mouse for experiments used the minimum number of the necessity. The mice were divided into each groups randomly. In this study, we have used humane endpoints for the infected animals. To determine when the animals should be euthanized, we have used the specific signs such as weight loss, inability to rise or ambulate, or dehydration. For euthanasia, cervical dislocation has been used by well-trained individuals. We have monitored the health condition of the animals at least twice a day. Pain relievers or anesthesia could not be administered in this study, because these treatments might influence the appearance of clinical signs such as progress of neurological symptoms or neuropathy derived from murine malarial infection.

### Induction of α-tocopherol deficiency

Plasma α-tocopherol deficiency was induced by treatment with probucol (Wako; Tokyo, Japan) in the diet. Six-week-old C57BL/6J mice were treated with 1% w/w probucol in the diet for 2 weeks. The control group was designated as C57BL/6J mice fed with a standard diet (75 mg/kg of α-tocopherol). After probucol treatment for 2 weeks, a subgroup of the treated mice was fed with a standard diet for 2 weeks. Blood samples were obtained under anesthesia with diethyl ether (Wako; Tokyo, Japan) by cardiac puncture using sodium citrate (Wako; Tokyo, Japan) as an anticoagulant, and then blood samples were centrifuged at 5000 rpm for 5 min at 4°C to remove the plasma and buffy coat [22].

### Experimental infections

Plasma α-tocopherol deficiency in mice was induced as described above. Then, mice were intraperitoneally inoculated with 0.2 mL of 1 × 10^5^ erythrocytes /mL infected with *Plasmodium yoelii* XL-17 [22]. Probucol treatment was continued throughout the experiments with infection in the probucol-treated group. Parasitemia, survival rate, and body weight were monitored every 2 days. In addition, hematological parameters such as erythrocyte count, hematocrit (HCT) percentage, and hemoglobin (Hb) concentration were determined. An additional infectious experiment was performed in order to obtain plasma and erythrocytes from infected and non-infected mice at different points after infection, namely days 4, 9, 12, 19, and 22 post-infection. In the second experiment with infection, the effect of 1% w/w probucol and dihydroartemisin (DHA) (Tokyo Chemical Industry Co.; Tokyo, Japan) was investigated following the protocol described by Gibbons *et al*. [29]. Briefly, α-tocopherol deficiency induction and infection were carried out as described above. Then, on days 3, 4, and 5 after infection, mice were treated with 30 mg/kg of DHA; thereafter, parasitemia and survival rate were monitored. In addition, mice were infected by *P*. *yoelii* XL-17 and simultaneously treated with 1% w/w probucol in the diet.

### Mosquito infection

Two C57BL/6J mice were infected with a frozen stock of *P*. *berghei* ANKA. Thereafter, female *Anopheles stephensi* were allowed to feed on anaesthetized *P*. *berghei* ANKA-infected C57BL/6J mice. After feeding, mosquitoes were kept at 19°C and 80% humidity. Twenty-one days after incubation, 5 mosquitos were placed in the presence of one probucol-treated mouse or a standard diet-fed mouse in individual cages. Thereafter, the mosquitos were allowed to blood feed for 30 min. In addition, the number of blood-feeding mosquitos was recorded. After blood feeding, parasitemia, survival rate, and clinical signs of cerebral malaria (CM) were monitored daily from both experimental groups.

### Detection of α-tocopherol by using high-performance liquid chromatography-electron capture detector (HPLC-ECD)

Plasma and erythrocyte α-tocopherol was extracted using a protocol described previously [30]. The erythrocyte samples were homogenized with saline (sample: saline 1: 3, w/w). Plasma samples and homogenized erythrocyte samples samples were extracted by chloroform/methanol (2/1 in volume) containing 100 μM butylated hydroxytoluene (BHT). Thereafter, the extracts of these samples were centrifuged at 15,000 rpm at 4°C. The concentration of α-tocopherol was determined by using an HPLC-ECD system with an electrochemical detector (NANOSPACE SI-1; Shiseido; Tokyo, Japan). The analyte was eluted with methanol/NaClO_4_ (50 mM) at a flow rate of 0.7 mL/min in a Wakosil-2 5C18 RS column (Wako; Tokyo, Japan). The concentration of α-tocopherol was determined by comparing the area under the curve of the analyte with that of the standard. A standard curve was prepared by using serial dilutions (1 μM, 500 nM, and 100 nM) of α-tocopherol standard (Eisai Chemical Company; Tokyo, Japan).

### Cholesterol measurements

Cholesterol concentration in plasma was determined by using the Cholesterol E-test (Wako; Osaka, Japan) according to the manufacturer’s instructions.

### Hematological determinations

Hematological parameters such as erythrocyte count, HCT value, and Hb concentration were determined. Briefly, 5 μL of blood taken from the tail was mixed with an isotonic buffer (Isotonac; MEK-510; NIHON KOHDEN; Tokyo Japan) and then analyzed using an automatic hematological analyzer (Celltac α, MEK−6358; NIHON KOHDEN; Tokyo Japan).

### Sample preparation for the analysis of lipid peroxidation products by LC-MS/MS and gas chromatography (GC)-MS

To evaluate the levels of lipid peroxidation products, the concentration of hydroxyoctadecadienoic acid (HODE) and 7β-hydroxycholesterol (7β-OHCh), which are generated from linoleic acid (LA) and cholesterol (Ch), respectively, was measured. To avoid the influence of the reduction of LA and Ch, the ratio of total HODE to LA (tHODE/LA) and the ratio of 7β-OHCh to total Ch (7β-OHCh/tCh) were measured [31,32].

13-Hydroxy-9Z, 11E-octadecadienoic acid (13-(Z,E)-HODE), 9-(Z,E)-HODE, and 13-HODE-d4 were obtained from Cayman Chemical Company (MI, USA); 9-(E,E)-HODE and 13-(E,E)-HODE were obtained from Larodan Fine Chemicals AB (Malmo, Sweden); 7β-OHCh was obtained from Steraloid Inc. (RI, USA); and the isotope 7β-OHCh-d7 was obtained from Medical Isotopes Inc. (NH, USA). Other materials were of the highest grade commercially available. In each point, 300 to 500 μL of blood was taken from abdominal aorta. Then plasma and erythrocytes were separated at 500 rpm for 5 min at 4°C. Then, erythrocytes were washed twice with a 3-fold volume of saline. Erythrocyte samples were extracted using a 4-fold volume of methanol that contained 100 μM 2,6-di-tertbutyl-4-methylphenol (BHT) by vortexing and centrifuging (20,400 × *g* at 4°C for 10 min). The data obtained from the erythrocyte analysis were normalized to the protein concentration measured with the BCAProtein assay kit (Thermo Scientific; Rockford, USA). Subsequently, 500 μL of methanol containing the internal standards 13-HODE-d4 (50 ng), 7β-OHCh-d7 (19 ng), and 100 μM BHT was added to 500 μL of erythrocyte sample or plasma sample, 50 μL of plasma mixed with 450 μL of saline. This was followed by reduction using triphenylphosphine (1 mM) at room temperature for 30 min. Next, the reduced sample was mixed with 1M KOH in methanol (500 μL) under a nitrogen atmosphere and incubated on a shaker for 30 min in the dark at 40°C. The mixture was acidified with 10% acetic acid in water (2 mL) and extracted with chloroform and ethyl acetate (chloroform: ethyl acetate = 4: 1, v/v, 5 mL). The sample was mixed using a vortex mixer for 1 min and centrifuged at 1750 × *g* for 10 min at 4°C. The chloroform and ethyl acetate layer was concentrated to around 1 mL after the removal of the water layer and divided equally into two portions.

### Analysis of HODE by LC-MS/MS

The divided chloroform and ethyl acetate solution was evaporated to dryness under nitrogen. The pelletedsample was reconstituted with methanol and water (methanol: water = 70: 30, v/v, 200 μL), and a portion of the sample (10 μL) was subjected to LC-MS/MS analysis. LC was carried out on an octadecyl-silica (ODS) column (Hypersil Gold, 3.0 μm, 100 × 2.1 mm; Thermo Fisher Scientific; CA, USA) in a column oven (CTO–20A; Shimadzu; Kyoto, Japan) set at 30°C. The LC apparatus consisted of an autosampler (SIL–20AC; Shimadzu; Kyoto Japan) and a pump (LC–20AB; Shimadzu; Kyoto Japan). The eluent condition was a gradient comprising solvent A (2 mM ammonium acetate in water) and solvent B (methanol: acetonitrile = 5: 95), at a flow rate of 0.2 mL/min. The initial composition of the gradient was 8% A and 20% B. The gradient was folded for 2 min and the composition was changed to 50% A and 50% B after 45 min. MS was carried out using a Thermo Finnigan TSQ Quantum Discovery Max, a triple-quadrupole mass spectrometer (Thermo Fisher Scientific; CA, USA) fitted with an electrospray ionization (ESI) source. ESI was carried out at a needle voltage of 4.2 kV. Nitrogen was used as the sheath gas (40psi) and auxiliary gas (12 units). The capillary was heated to 270°C, and the spectrometers were optimized to achieve the maximum sensitivity. A specific precursor-to-product ion transition was carried out by selected reaction monitoring (SRM) after collision-induced dissociation in the negative mode. Argon was used as the collision gas, and the collision cell pressure was 1.5 mTorr. The precursor, product ions, and collision energy were determined after the optimization of MS/MS as follows: m/z = 295.0 and 194.6–195.6 at 21 eV for both 13-(Z,E)-HODE and 13-(E,E)-HODE, m/z = 295.0 and 170.5–171.5 at 24 eV for both 9-(E,Z)-HODE and 9-(E,E)-HODE, and m/z = 299.0 and 197.6–198.6 at 15 eV for 13-HODE-d4.

### Analysis of 7β-OHCh, Ch, and LA by GC-MS

The other portion of the chloroform and ethyl acetate solution was also evaporated to dryness under nitrogen. A silylating agent, N,O-bis(trimethylsilyl)-trifluoroacetamide (BSTFA, 50 μL), was added to the dried residue. The solution was vigorously mixed by vortexing for 0.5 min and incubated for 60 min at 60°C to obtain trimethylsilyl esters and ethers. An aliquot of this sample was injected into a gas chromatograph (GC 6890 N; Agilent Technologies; Palo Alto, CA, USA) that was equipped with a quadrupole mass spectrometer (5973 Network; Agilent Technologies). A fused-silica capillary column (HP-5MS; 5% phenyl methyl siloxane, 30 m × 0.25 mm; Agilent Technologies) was used. Helium was used as the carrier gas at a flow rate of 1.2 mL/min. Temperature programming was carried out from 60°C to 280°C at 10°C/min. The injector temperature was set at 250°C, and the temperatures of the transfer line to the mass detector and ion source were 250°C and 230°C, respectively. The electron energy was set at 70 eV. 7β-OHCh, Ch, and LA were identified on the basis of their retention times and mass patterns; ions having m/z = 456 for 7β-OHCh, 458 for Ch, and 337 for LA were selected for the quantification. 7β-OHCh, Ch, and LA were identified quantitatively by using 7β-OHCh-d7 as the internal standard.

### Analysis of the α-tocopherol transfer protein (*α-ttp*) gene in the liver by real-time quantitative polymerase chain reaction (qPCR)

The mRNA expression of *α-ttp* was assessed by real-time qPCR. Liver samples were obtained from uninfected C57BL6J mice (n = 3) and 1% w/w probucol-treated mice (n = 6). In addition, samples from probucol-treated mice infected with *P*. *yoelii* XL-17 were obtained at different points after infection (i.e., day 4, 9, 12, 19, and 22). Briefly, a portion of the liver was removed aseptically, placed immediately in liquid nitrogen, and then stored at −80°C. For RNA extraction, tissues were homogenized by using a Biomasher (Funakoshi; Tokyo, Japan). Total RNA was extracted using the TRI reagent kit (Sigma; St. Louis, MO). The concentration of total RNA was adjusted to a final concentration (50 ng/μL). Thereafter, real-time qPCR was performed using specific double-labeled probes employing an ABI PRISM 7900 HT Sequence detection System (Applied Biosystems; California, USA). The primers and probes used are shown in S1 Table. The PCR reaction mixture and mRNA amplification conditions used in this study are described in a previous study [22]. Briefly, the reaction mixture (20 μL) for mRNA amplification consisted of 10 μL of 2 × master mix without uracil N-glycosylase (UNG), 0.5 μL of 40 × multiscribe and RNase inhibitor mix, 1 μL of 2 × TaqMan Gene expression assays, 4 μL of total RNA template (50 ng/μL), and 4.5 μL of RNase-free double-distilled water. At first, the template was reverse-transcribed at 48°C for 30 min and then denatured at 95°C for 10 min with both reactions taking place in one cycle. The next step involved 45 cycles of amplification reactions at 95°C for 15 s and 60°C for 1 min. Standard curves were prepared by using a predetermined concentration of serially diluted total RNA obtained from the livers of uninfected C57BL/6J mice. To enable the normalization of relative mRNA expression of the target genes, *β-actin*, *glyceraldehyde-3-phosphate dehydrogenase* (*GAPDH*), and *18SrRNA* were included as internal control genes (S1 Table). Normfinder software was used to select the best internal control gene. Then, the relative expression of each gene was normalized with the best-selected internal control gene.

### Analysis of the antioxidant enzymes from *P*. *yoelii XL-70* by real-time qPCR

The mRNA expression of *1-Cys peroxiredoxin* (*Prx*) and *thioredoxin peroxidase* (*Tpx-1*), which are antioxidant enzymes of the parasites [33,34], and *Hsp-70*, which was reported to ameliorate the toxic effects of the oxidative stress [35], were measured for investigation the parasite response against oxidative stress. Blood was collected from mice fed with standard diet (n = 6) and mice fed with probucol diet (n = 6) and mixed with an RNA isolation buffer (RNA later; AMBION; Austin, Texas) to prevent RNA degradation. Then, the samples were centrifuged at 15,000 rpm for 3 min at 4°C, the supernatant was extracted, and total RNA was extracted using the Mouse RiboPure-Blood RNA isolation kit (AMBION; Austin, Texas). RNA quality was assessed by using the Experion Automated Electrophoresis System (ExperionRNA StdSens Analysis Kit; Bio-Rad; Hercules, CA). Then, real-time qPCR was performed. Briefly, the reaction mixture (20 μL) consisted of 10μL of EXPRES SYBR^R^ GreenERqPCR Supermix Universal, 0.4 μL of each primer, 0.4 μL of ROX reference dye, 0.5 μL of EXPRESS Superscript^R^ Mix for One-Step SYBRR GreenER, 5 μL of template RNA, and 3.5 μL of diethylpyrocarbonate (DEPC)-treated water. First, the template was reverse-transcribed at 48°C for 30 min and then denatured at 50°C for 5 min followed by exposure at 95°C for 2 min. The next step involved 40 cycles of amplification reactions at 95°C for 15 s, and 60°C for 1 min, followed by a melting curve analysis. Then, a standard curve was prepared using a predetermined concentration of serially diluted total RNA obtained from infected blood. The relative mRNA expression of the target genes was normalized against *18S rRNA* (S1 Table).

### Statistical analysis

Statistical analyses were performed by analysis of variance (ANOVA) by using SPSS version 21.0, and a *p*-value of less than 0.05 was considered significant. For the survival rate analysis, the Kaplan–Meier long-rank method was performed. A *p* value less than 0.05 was considered statistically significant.

## Results

### Probucol pre-treatment before infection conferred a protective effect against *P*. *yoelii XL-17* and *P*. *berghei* ANKA infections

**Fig 1:**
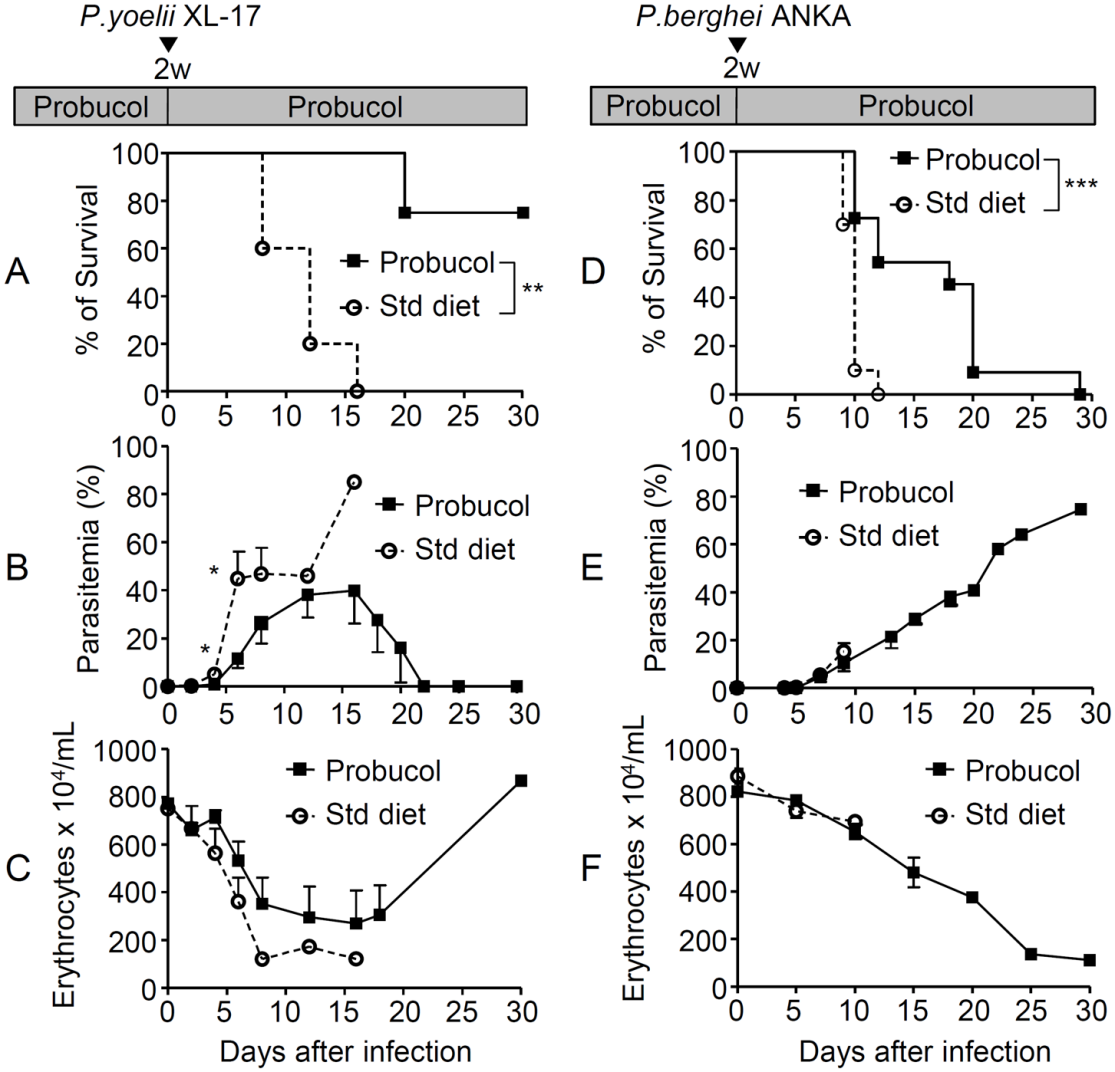
Probucol treatment confers protection to mice against *Plasmodium yoelii* XL-17 infection. Six-week-old C57BL/6J mice were treated with a standard diet (Std) or 1% w/w probucol in the diet for 2 weeks and then infected with 0.2 mL of 1 × 10^5^ erythrocytes /mL infected with *P*. *yoelii* XL17. Survival (A), mean parasitemia (B) and the erythrocyte count (C) of probucol-treated mice (n = 4) and Std diet-fed mice (n = 5) was monitored. Probucol treatment extended the survival rate after infection with *P*. *berghei* ANKA. Six-week-old C57BL/6J mice were treated with Std diet or 1% w/w probucol in diet for 2 weeks and then infected with *P*. *berghei* ANKA through mosquito bite. Survival (D) and mean parasitemia (E) of probucol-treated mice (n = 11) and Std diet-fed mice (n = 10). Parasitemia was monitored every 2 days and the erythrocyte count was determined (F). All data are expressed as mean ± standard error (SE). Statistical analysis was carried out by analysis of variance (ANOVA). For the survival rate analysis, the Kaplan–Meier long-rank method was performed; *p < 0.05, **p < 0.025, and ***p < 0.001 compared to standard diet-fed mice.

Seventy-five percent of the mice treated with probucol survived after *P*. *yoelii* XL-17 infection, whilst all non-treated mice died by day 16 post-infection (Fig 1A). Significantly low parasitemia was observed in the treated mice compared to non-treated mice throughout infection (Fig 1B). Parasitemia in the treated mice increased until day 16 post-infection. Then, it started to decrease and parasites were completely cleared by day 25 post-infection.

Anemia was evident in both control and experimental groups (Fig 1C). However, probucol-treated mice recovered from anemia. The number of erythrocytes in probucol treated mice significantly decreased from day 8 to 16 post-infection and returned to normal levels at day 18 post-infection.

The median survival in mice treated with probucol and infected with *P*. *berghei* ANKA was 18 days whilst in non-treated mice it was 10 days (Fig 1D). The parasitemia of non-treated mice was slightly higher than that of probucol-treated mice without significant difference (Fig 1E). Interestingly, probucol-treated mice that survived longer than 10 days died with anemia and without clinical signs of cerebral malaria. However, all non-treated mice died showing clinical signs of cerebral malaria, such as paralysis, convulsions, stupor, and rolling over (Fig 1F) [36].

### Plasma α-tocopherol and cholesterol levels decreased after probucol treatment

**Fig 2:**
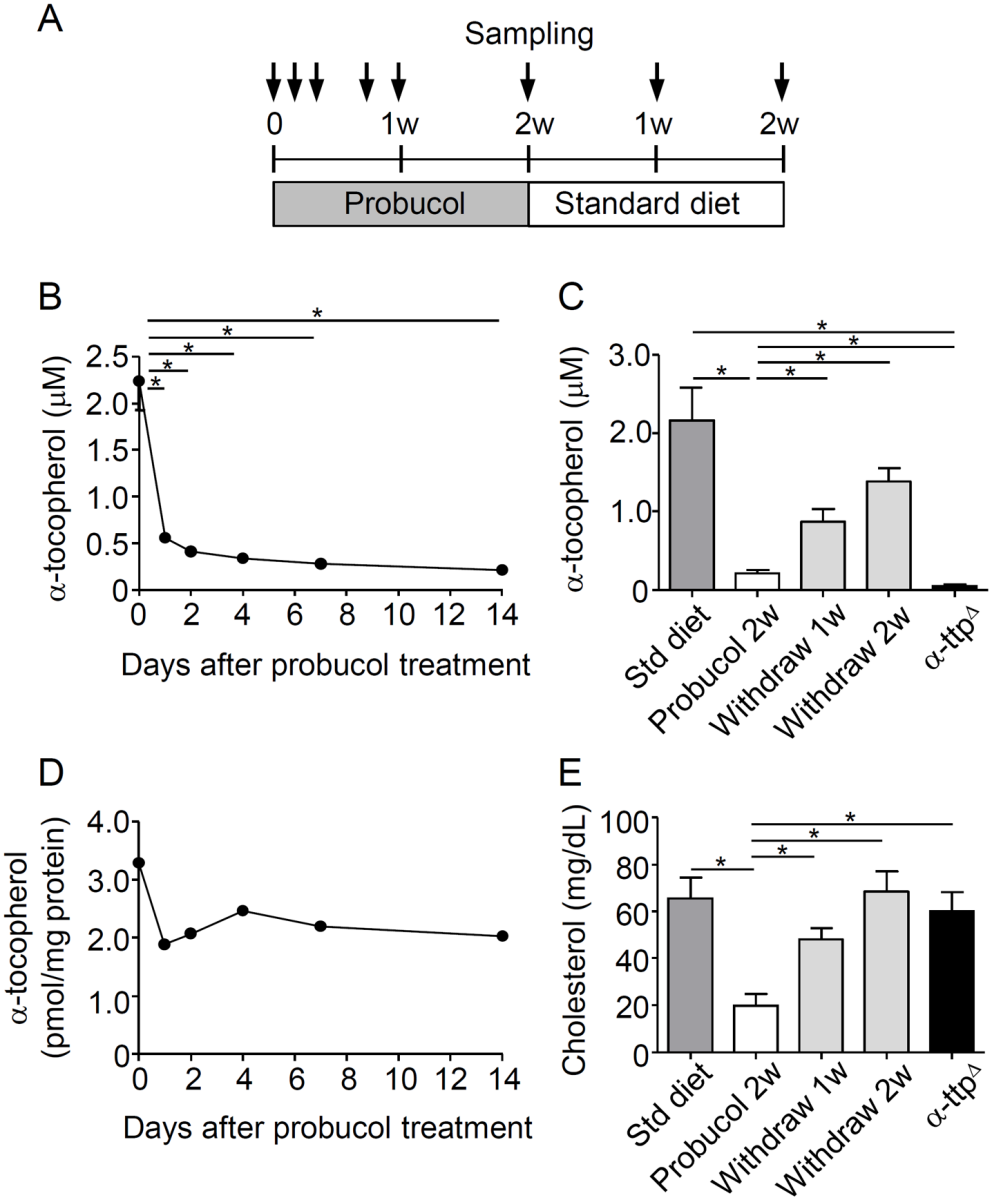
Plasma concentrations of α-tocopherol and cholesterol decreased after probucol treatment. A, Experimental design for α-tocopherol deficiency induction using 1% w/w probucol in the diet. Six-week-old C57BL/6J mice were treated with 1% w/w probucol for 2 weeks. Then, mice were withdrawn from the probucol diet and changed to a standard diet for 2 weeks. Plasma and erythrocyte samples were obtained at day 0, 1, 2, 4, 7, and 14 after starting probucol treatment (n = 5 per group) and 1 or 2 weeks post-withdrawal (n = 3 per group). In addition, the plasma of eight-week-old α-tocopherol transfer protein knockout (α-ttp^Δ^) mice fed a standard diet was obtained (n = 3). Plasma α-tocopherol concentrations were measured after probucol treatment (B) and after withdrawal (C). The levels of α-tocopherol in erythrocytes were normalized with the protein concentration (n = 5 per group) (D). Plasma cholesterol concentrations were measured by using the cholesterol E-test after 2 weeks of probucol treatment and after withdrawal (n = 3) (E). All data are expressed as mean ± SE. Statistical analysis was carried out by analysis of variance (ANOVA; multiple comparisons Tukey’s test). *p < 0.05.

The kinetics of α-tocopherol and cholesterol concentrations during probucol treatment and after withdrawal are shown in Fig 2A. Probucol treatment dramatically reduced the plasma concentration of α-tocopherol to 25% ± 0.5% and to 9.2% ± 1.3% of the control levels after one day and 2 weeks of treatment, respectively (Fig 2B). At 2 weeks after probucol withdrawal, the concentration of α-tocopherol reached 67.1% ± 34.6% of the levels observed in mice fed with a standard diet (Fig 2C). In addition, plasma concentration of α-tocopherol in α-ttp^Δ^ mice fed with standard diet was lower than that of mice treated with probucol for 2 weeks. The concentration of α-tocopherol in erythrocytes did not significantly reduce after probucol treatment for 2 weeks (Fig 2D).

At 2 weeks after treatment with probucol, cholesterol concentration significantly decreased to 30.1% of the control levels (19.7 mg/dL ± 5.2 mg/dL vs. 65.6 mg/dL ± 8.8 mg/dL) (Fig 2E). Nevertheless, 2 weeks after probucol withdrawal, the concentration of cholesterol returned to the control level (68.5 mg/dL ± 8.6 mg/dL).

Since diet-induced α-tocopherol deficiency has been associated with anemia [37], hematological parameters were monitored under probucol treatment. Anemia was not observed at 2 weeks after probucol treatment. The number of erythrocytes (759.7×10^4^ /mL ± 33.9×10^4^ /mL), hemoglobin (Hb) concentration (11.5 mg/dL ± 0.5 mg/dL), and hematocrit (HCT) percentage (36.0% ± 1.4%) in probucol-treated mice were similar to those observed in non-treated mice (773.5×10^4^ /mL ± 11.4×10^4^ /mL, 11.9 mg/dL ± 0.3 mg/dL, and 35.8% ± 0.7%, respectively).

### Survival rate was significantly extended in mice infected with *P*. *yoelii* XL-17 and simultaneously treated with probucol

**Fig 3:**
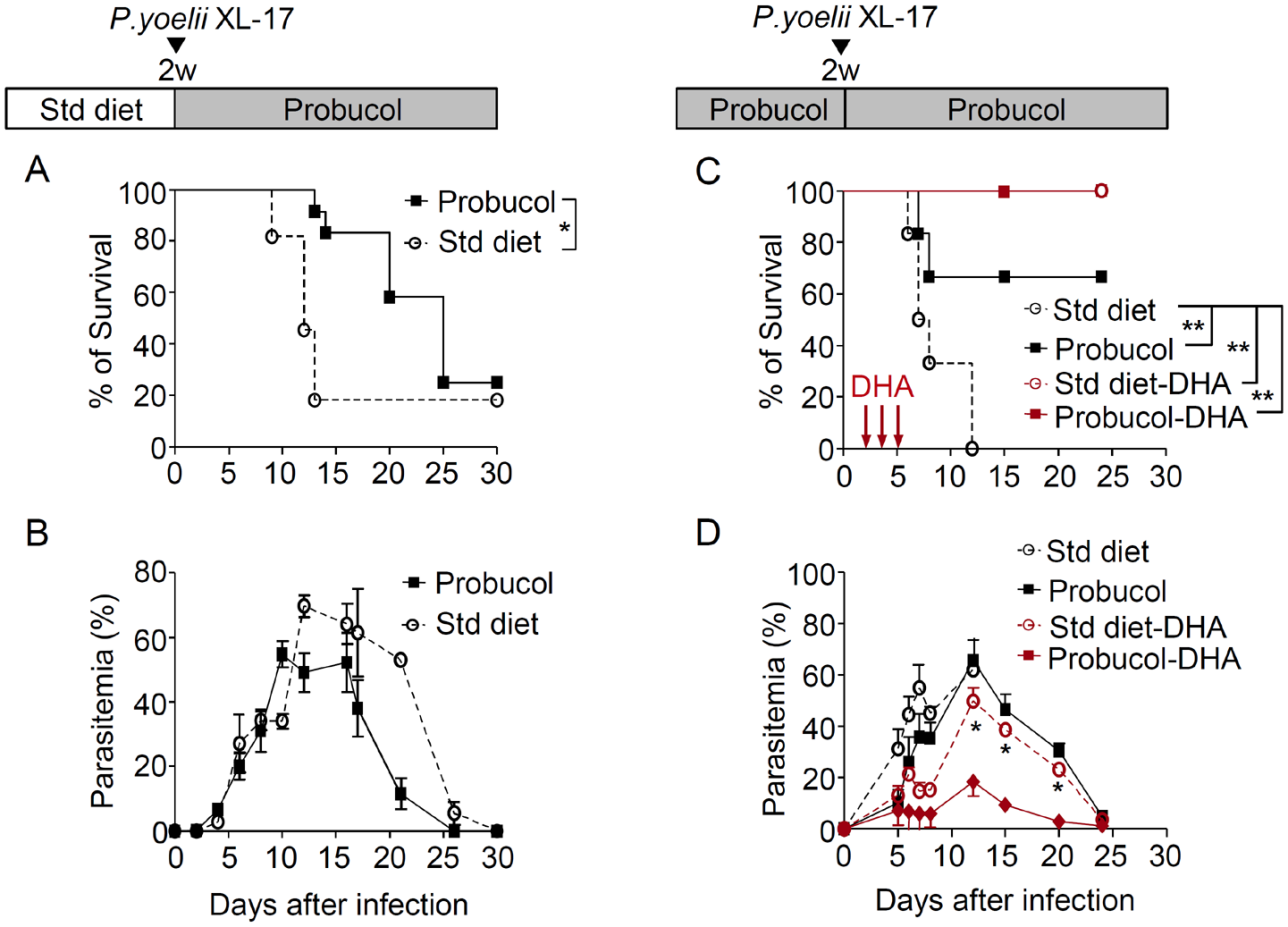
Effect of simultaneous probucol treatment and infection and effect of probucol in combination with antimalarial. The survival was improved in mice infected with *Plasmodium yoelii* XL-17 and subjected to simultaneous probucol treatment. Eight-week-old mice fed a standard (Std) diet were infected with *P*. *yoelii* XL-17. Probucol (1% w/w) started simultaneously with the infection (n = 12). The control group fed Std diet (n = 11). Survival (A) and parasitemia (B) were monitored. Parasite proliferation was inhibited by combination therapy with probucol and DHA. Six-week-old mice pre-treated with Std or probucol diet for 2 weeks were infected with *P*. *yoelii* XL-17. Thereafter, mice were treated with DHA (30 mg/kg) or PBS on day 3, 4, and 5 post-infection. Survival (C) and parasitemia (D) of mice treated with Std diet with/without DHA and probucol with/without DHA, (n = 6 per group) were monitored. Data are expressed as mean ± SE. Statistical analysis was performed by ANOVA and Kaplan-Meier log-rank method. *p < 0.05, **p < 0.025.

The plasma level of α-tocopherol significantly reduced at 1 day after probucol treatment. Therefore, probucol treatment was started immediately after *P*. *yoelii* XL-17 inoculation. The survival rate was significantly extended in probucol-treated mice (Fig 3A). However, parasitemia kinetics were similar among the control and experimental groups (Fig 3B).

### Combination therapy with probucol enhanced the protective effect of dihydroartemisin

The beneficial effect of the combined treatment with DHA and probucol was evident against *P*. *yoelii* XL-17 infection (Fig 3C and 3D). In this experiment, to reach sufficient reduction of plasma α-tocopherol concentration, mice were inoculated with the parasite after 2 weeks pre-treatment of probucol. No mortality was observed in both the combined therapy group and the singly DHA-treated group (Fig 3C). Nonetheless, mice treated with probucol plus DHA showed significantly lower parasitemia from day 12 after infection than those treated only with DHA (Fig 3D).

### The ratios of lipid peroxidation products to parent lipids changed in plasma and Eythrocytes upon probucol treatment and after *P*. *yoelii* XL-17 infection

**Fig 4:**
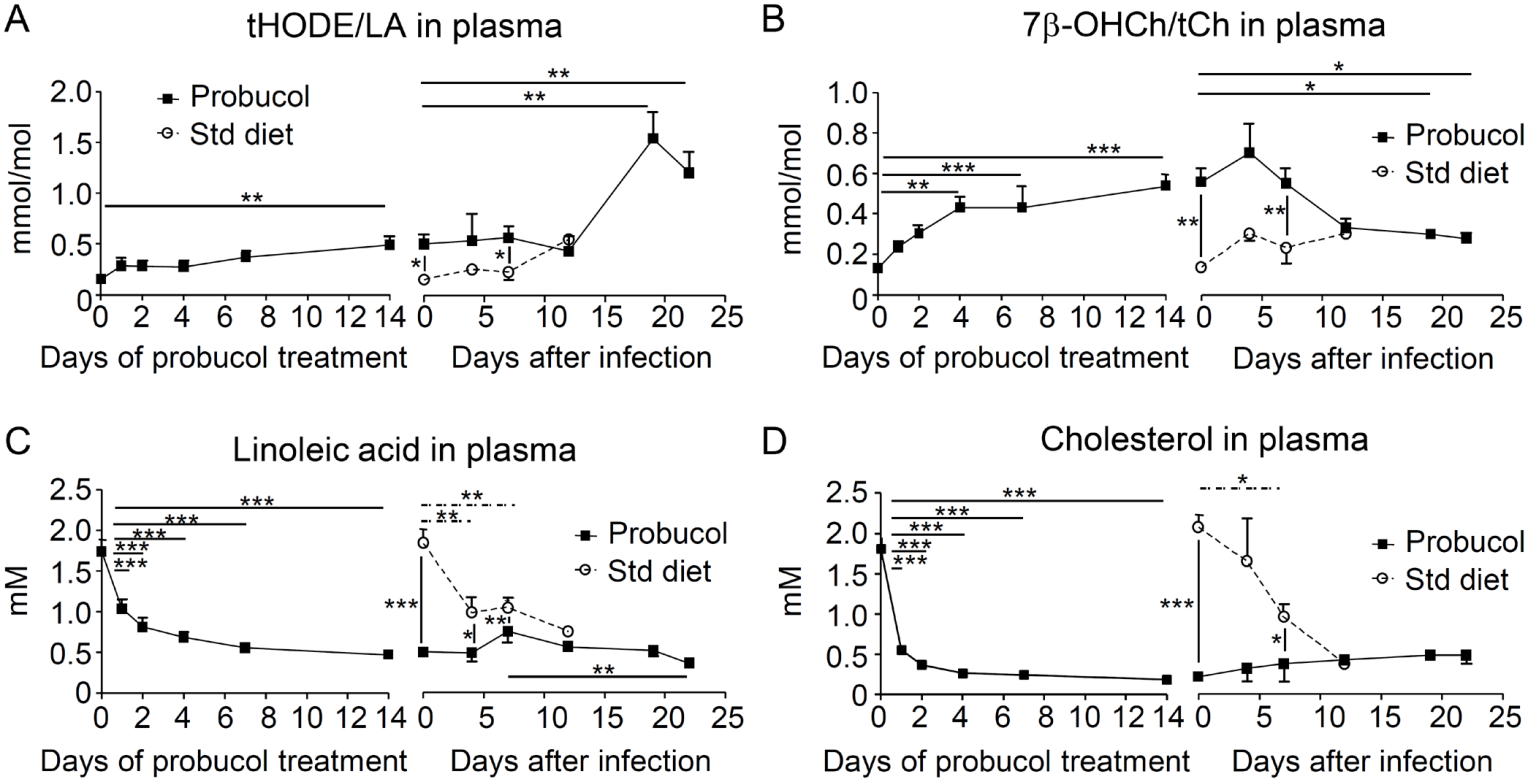
The ratios of lipid peroxidation products to parent lipids in plasma increased after probucol pre-treatment. Six-week-old C57BL/6J mice were treated with 1% w/w probucol in the diet for 2 weeks and then infected with 0.2 mL of 1 × 10^5^ erythrocytes /mL infected with *Plasmodium yoelii* XL-17. Plasma samples were obtained at day 0, 1, 2, 4, 7, and 14 after starting the probucol diet (n = 5 per group) and at day 0, 4, 7, 12, 19, and 22 post-infection (n = 2 to 7). The ratio of total hydroxyoctadecadienoic acid (HODE), a peroxidation product of linoleic acid (LA), to linoleic acid (tHODE/LA) in plasma (A) and the ratio of 7β-hydroxycholesterol (7β-OHCh), a peroxidation product of cholesterol, to total cholesterol (7β-OHCh/tCh) in plasma (B) were measured. The concentration of LA (C) and tCh (D) were measured by using gas chromatography-mass spectrometry (GC-MS). All data are expressed as mean ± SE. Statistical analysis was carried out by analysis of variance (ANOVA). *p < 0.05, **p < 0.025, and ***p < 0.001. The solid barsindicate the significant changes in probucol-treated groups and the dotted bars indicate the significant changes in standard (Std) diet-fed mice.

The ratios tHODE/LA and 7β-OHCh/tCh significantly increased after 2 weeks of probucol treatment (152.1 μmol/mol ± 11.8 μmol/mol vs. 490 μmol/mol ± 80.9 μmol/mol and 131.1 μmol/mol ± 14.0 μmol/mol vs. 538.2 μmol/mol ± 58.5 μmol/mol, respectively) (Fig 4A and 4B). After infection, the increments of change in the ratios of these lipid peroxidation products to each parent lipid the plasma were observed (Fig 4A). The plasma level of tHODE/LA significantly increased in probucol-treated mice on day 19 post-infection (Fig 4A). By contrast, the plasma level of 7β-OHCh/tCh significantly decreased after infection in probucol-treated mice, whilst that in non-treated mice remained stable (Fig 4B). Remarkably, the levels of both oxidation products were higher until day 7 post-infection in probucol-treated mice than those in non-treated mice (Fig 4A and 4B).

LA and tCh concentrations in probucol-treated mice were significantly lower than those observed in non-treated mice (Fig 4C and 4D); however, their concentrations significantly decreased in non-treated mice after infection (Fig 4C and 4D). In probucol-treated mice, LA concentration significantly decreased on day 20 after infection (Fig 4C), whilst that of tCh remained unchanged (Fig 4D).

**Fig 5:**
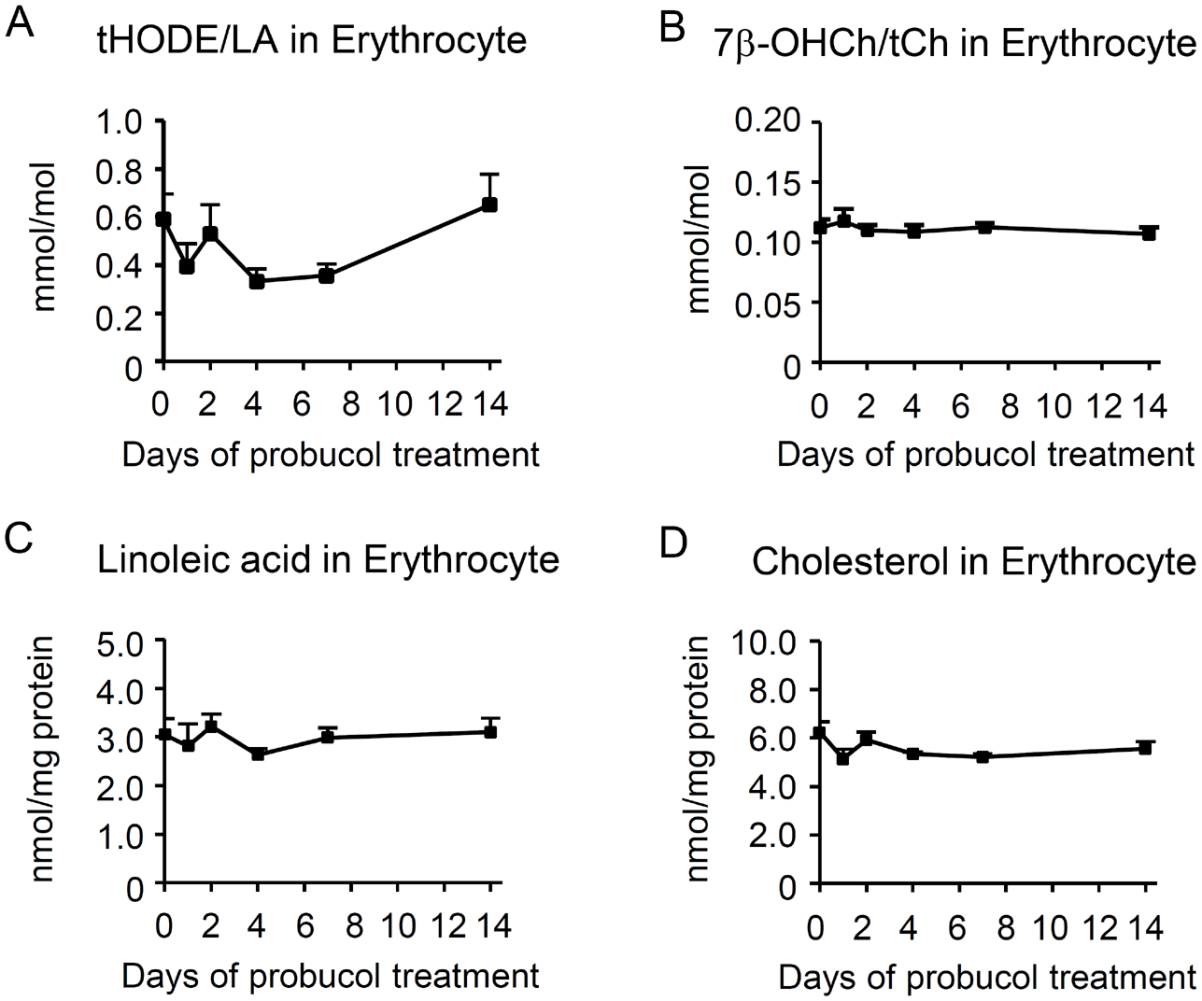
Probucol pre-treatment did not increase the lipid peroxidation products in erythrocytes. Six-week-old C57BL/6J mice were treated with 1% w/w probucol in the diet for 2 weeks and then infected with 0.2 mL of 1 × 10^5^ erythrocytes /mL infected with *Plasmodium yoelii* XL-17. Erythrocyte samples were obtained from 0, 1, 2, 4, 7, and 14 days after starting the probucol diet (n = 5 per group). The ratio of total hydroxyoctadecadienoic acid (HODE), a peroxidation product of linoleic acid (LA), to linoleic acid (tHODE/LA) in erythrocytes (A) and the ratio of 7β-hydroxycholesterol (7β-OHCh), a peroxidation product of cholesterol (Ch), to total cholesterol (7β-OHCh/tCh) in erythrocytes (B) were measured. Moreover, the concentrations of LA (C) and Ch (D) in erythrocytes were determined by normalizing by the protein concentration. Statistical analysis was carried out by analysis of variance (ANOVA). *p < 0.05 and **p < 0.025.

By contrast, probucol treatment for 2 weeks did not increase the levels of tHODE/LA and 7β-OHCh/tCh in erythrocytes (Fig 5A and 5B).

The concentrations of LA and tCh did not change in erythrocytes upon probucol treatment for 2 weeks (Fig 5C and 5D).

### Plasma α-tocopherol concentration decreased upon downregulation of *α-ttp* after infection

**Fig 6:**
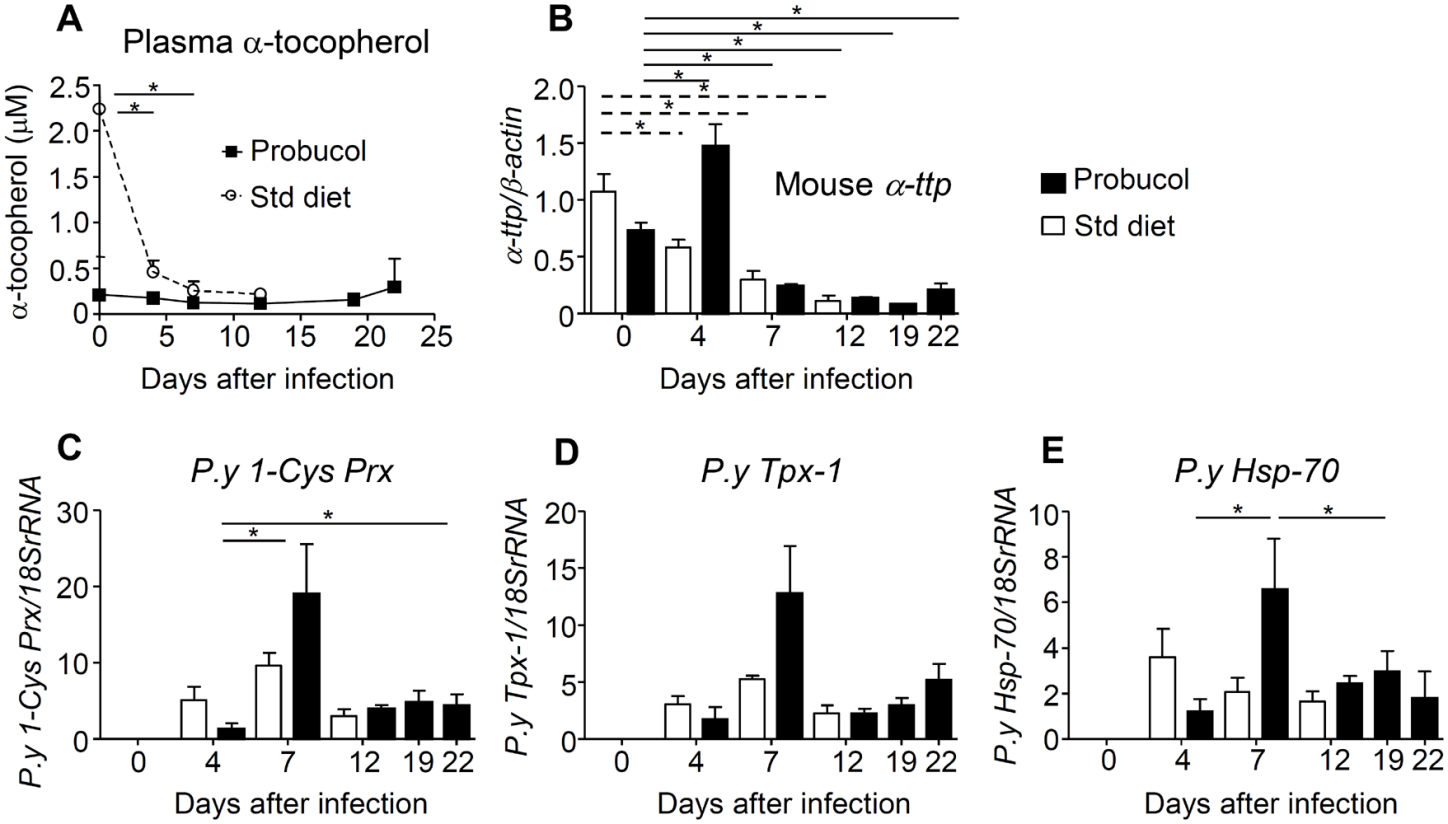
Oxidative response of mice and parasites to α-tocopherol deficiency after infection. Six-week-old C57BL/6J mice were treated with 1% w/w probucol in the diet for 2 weeks and then infected with 0.2 mL of 1 × 10^5^ erythrocytes infected with *Plasmodium yoelii* XL-17. Plasma, total blood, and liver samples were obtained on day 0, 4, 7, 12, 19, and 22 post-infection (n = 2 to 7). The plasma level of α-tocopherol (A) and the mRNA expression of *α-ttp* in liver (B) were analyzed. The mRNA expression of the parasite antioxidant enzymes, *P*.*y 1-Cys Prx* (C) and *P*.*y Tpx-1* (D), and the oxidative stress response protein *P*.*y Hsp-70* (E) were analyzed using parasite-specific primers and probes. All data are expressed as mean ± SE. Statistical analysis was carried out by analysis of variance (ANOVA). *p < 0.05.

After infection with *P*. *yoelii* XL-17, plasma concentration of α-tocopherol of non-treated mice significantly decreased from the pre-infection levels (2.16 μM ± 0.42 μM vs. 0.25 μM± 0.09 μM). However, it did not change in probucol-treated mice (0.21 mM ± 0.03 mM vs. 0.29 mM ± 0.30 mM) (Fig 6A). Thus, we further explored the mechanism of reduction of α-tocopherol in plasma after infection. The mRNA expression of *α-ttp* in the liver significantly decreased in non-treated mice after infection (Fig 6B). However, in probucol-treated mice, its expression was significantly upregulated only at day 4 post-infection, while afterward it was significantly downregulated.

### Antioxidant enzymes in parasites were upregulated in the treated mice after infection

The mRNA expressions of *1-Cys Prx* and *Tpx-1* of the parasite were significantly upregulated at day 7 post-infection in probucol-treated mice, while afterward mRNA expression was significantly downregulated (Fig 6C and 6D). Interestingly, the mRNA expression of both enzymes showed the tendency to increase in probucol-treated mice at day 7 post-infection. The mRNA expression of *Hsp-70* showed a similar expression pattern to *1-Cys Prx* in probucol-treated mice (Fig 6E).

## Discussion

In the present study, the antimalarial effects of the reduction of α-tocopherol levels induced by probucol treatment were characterized. Strikingly, probucol pre-treatment conferred protection against *P*. *yoelii* XL-17 infection. Combination therapy with probucol enhanced the protective effect of DHA. The efficacy of probucol in decreasing the concentration of α-tocopherol in plasma was evident, even after one day of treatment. Moreover, 14 days after probucol treatment, the ratio of lipid peroxidation products to parent lipids increased in plasma but not in erythrocytes.

During malaria infection, host and parasites are under high oxidative stress due to hemoglobin consumption, which leads to the production of high quantities of reactive oxygen species (ROS) [16]. The increment of tHODE/LA and 7β-OHCh/tCh in plasma after infection (Fig 4A and 4B) which were observed in standerd diet-fed mice indicated the infection-induced oxidative stress. The decline of 7β-OHCh/tCh observed in probucol-fed mice after infection (Fig 4B) might be induced by the protective effect against infection. However, the mechanism of the increment of tHODE/LA observed in probucol-fed mice after infection (Fig 4A) remains unclear.

*Plasmodium* parasites are highly susceptible to oxidative injury, because they lack glutathione peroxidase and catalase, the two major antioxidant enzymes in eukaryotic cells [38]. Thus, it is suggested that *Plasmodium* uses members of the peroxiredoxin family as the principal antioxidant enzymes to reduce peroxides, whose levels increase in infected erythrocytes during parasite development [17]. It has been reported that mice fed with α-tocopherol-deficient diets showed resistance against malaria infection [39]. The mechanism proposed for this resistance is the increased susceptibility of the parasite to oxidative injury in the absence of α-tocopherol, which is an important free radical scavenger. In fact, we previously reported that the proliferation of *P*. *yoelii* XL-17 and *P*. *berghei* NK65 was dramatically inhibited in α-ttp^Δ^mice whose α-tocopherol level in plasma is undetectable [21]. We have also demonstrated that this protective effect can be reversed by feeding α-ttp^Δ^mice with α-tocopherol-supplemented diets [21,22]. Together, this evidence clearly indicates that α-tocopherol deficiency in the host generates an environment of high oxidative stress and confers resistance against murine malaria. In the present study, the significant increment of the ratio of lipid peroxidation products to parent lipids in plasma after 2 weeks of probucol pre-treatment (Fig 4) demonstrated that probucol modifies the redox environment. Since the levels of lipid peroxidation product should strongly correlate with the levels of oxidative stress, the oxidative stress was evaluated by both tHODE/LA and 7β-OHCh/tCh levels in this study. Althogh the plasma oxidation level was elevated by probucol treatment, the oxidation level in erythrocyte could be independent of that in plasma. Thus, it might be essential to evaluate by an additional method which can directly observe oxidative stress inside erythrocyte, such as measurement using a fluorescence indicator. However, such assessment could not be applied in this study, because of technical difficulties. Instead, we confirmed increasing of mRNA levels in *1-Cys Prx* and *Tpx-1* of parasites within erythrocytes. The significant upregulation of *1-Cys Prx*, *Tpx-1*, and *Hsp-70* in *P*. *yoelii* (Fig 6) suggested that the parasites faced an oxidative stressful environment after infection. In addition, the significant downregulation of the mRNA expression of *α-ttp* in non-treated mice, which is directly related with the significant reduction of the concentration of α-tocopherol in plasma, might suggest the attempts of the host to reduce the concentration of α-tocopherol in circulation and thus prevent parasite proliferation (Fig 6). The mechanism of *α-ttp* upregulation in probucol-treated mice at day 4 post-infection remains unclear.

It has been reported that oxidized polyunsaturated fatty acids had a parasiticidal effect. Kumaratilake *et al*. [40] demonstrated that oxidized docosahexaenoic acid (C_22:6,n-3_) and arachidonic acid (C_20:4,n-6_) inhibit the growth of *P*. *falciparum* in erythrocyte. This antiparasitic activity of oxidized fatty acids was reduced when parasitized erythrocyte had been preincubaed with antioxidant, butylated hydroxytoluene (BHT) or α-tocopherol, and then exposed to the fatty acids. These results that malaria parasite was vulnerable to lipid oxidation products support our present results.

Unexpectedly, the increment of the ratio of lipid peroxidation products to parent lipids in erythrocytes, in which malaria parasites develop, was undetectable. Thus, it seems that the oxidative stress in plasma is more harmful to malaria parasites than that in erythrocytes. It has been reported that *P*. *falciparum* parasites invade erythrocytes and immediately modify their membrane [41,42]. Moreover, these alterations facilitate the movement of nutrients and waste products into and from erythrocytes, respectively. In addition, malaria parasites can uptake nutrients from the environment through ion channels [43] and tubovesicular membrane networks extending from the parasite vacuole membrane [41,42,44]. Thus, the active interaction between parasite and environment is evident and suggests that ROS and oxidation products in plasma reach malaria parasites inside erythrocytes and damage them. Blood-stage plasmodium parasite is reported to require high-density lipoprotein (HDL) particles for source of cholesterol [45,46]. On the other hand, probucol has a reduction effect of the HDL concentration in circulation via inactivation of ABCA1 transporter [25,26]. In summary, probucol pre-treatment disrupts the redox balance and reduces plasma lipid nutrients such as tCh and LA (Fig 4), which are essential for parasite proliferation after invading the erythrocytes. Therefore, both events may affect the proliferation of the parasites or make the parasites more susceptible to the oxidative damage.

The fact that simultaneous probucol treatment and parasite infection could not rescue the mice from death but significantly improved their survival may suggest that both α-tocopherol concentration and the redox status are important determinants for parasite proliferation. In addition, probucol treatment significantly improved the survival rate of mice infected with *P*. *berghei* ANKA and the death of these mice was attributed to anemia. This result suggests that it is possible that probucol treatment exerts other functions related to the modulation of the anti-inflammatory response of the host [47]. Moreover, the present study demonstrated that probucol treatment in combination with DHA reduces parasite proliferation (Fig 3). These results suggest that the dosages of DHA can be decreased when they are combined with probucol. Moreover, these combinations might reduce the toxic effects of these agents and suppress the appearance of resistance strains against these antimalarial agents. Because probucol affects malaria parasites indirectly, it is presumed that it will be difficult for malaria parasites to acquire resistance against it.

The probucol dosage used in this study converted by the body surface area was 5.3 times higher than the maximum dosage used in the treatment of hyperlipidemia [47]. The oral 50% lethal dose (LD50) of probucol in rats and mice was reported to be higher than 5000 mg/kg body weight [48]. And the clinical maximum dose of probucol for hyperlipidemia therapy is 1000 mg/day. If average human body weight is 50 kg, the clinical dose of probucol becomes 20 mg/kg body weight. In this study, the dose of probucol to mice was estimated about 1200 mg/kg body weight. Thus, the dose of probucol used in this study was higher than the clinical dose, however it was lower than the LD50. It has been reported that the adverse effects of probucol, such as ventricular arrhythmias including “torsades de pointes” and prolongation of the QT interval, may occur [49,50]. Nevertheless, in this study, mice treated with 1% w/w dietary probucol did not show unexpected death, anemia, or any other side effects, including body weight loss.

Chronic α-tocopherol deficiency is reported to be associated with ataxia [51,52]. In order to develop such neurological abnormalities, the organism is required to be in a long-term α-tocopherol-deficient state. The present study showed that probucol can induce a temporary α-tocopherol deficiency, but it also showed that plasma α-tocopherol concentration was quickly recovered after withdrawal (Fig 2C). Indeed, probucol treatment does not lead to a chronic α-tocopherol deficiency. Further studies are required to adjust the appropriate dosage of probucol for malaria treatment in humans.

Probucol is used currently as a therapeutic agent for hyperlipidemia in several countries, including Japan [53]. However, probucol was withdrawn from use in most of the Western countries after statins, HMG-CoA reductase inhibitors, appeared on the market in 1987 in USA and in 1989 in Japan. However, probucol has continued to reveal its clinical benefits and unique mechanism of action in various clinical studies as well as basic researches in all over the world. Recent scientific data and findings in clinical development have supported probucol’s reposition as a therapeutic drug and the need for a reappraisal of this drug. In this paper, we rather emphasized a potential application to treat malaria using existing drugs of which safety and pharmacokinetic effects have been confirmed already. As the malaria is the disease threatening the developing countries, we need to find out a method to develop a new anti-malaria drug with substantially low-cost.

New strategies for the treatment of malaria are desperately needed; probucol might be a candidate for drug against malaria infection by inducing α-tocopherol deficiency without using α-ttp gene mutation or dietary α-tocopherol restriction. It is possible that a drug re-profiling of probucol will help discover an affordable new class of medicine fighting malaria. The present study opens several new directions for future research. Admittedly, the results of animal experiments cannot be easily extrapolated to humans, but this study implies a potential benefit of probucol against malaria infection.

## Supporting Information

S1 List The ARRIVE Guidelines Checklist. (PDF)

S1 Table Nucleotide sequences of primers and probes used for real time-quantitative PCR. (PDF)
