# Supplementary material for: Probucol-Induced α-Tocopherol Deficiency Protects Mice against Malaria Infection
Source: PLoS One. 2015 Aug 21;10(8):e0136014. doi: 10.1371/journal.pone.0136014 (PMC4546625; doi:10.1371/journal.pone.0136014)
Supplement: S1 Table — (PDF) [file pone.0136014.s002.pdf]

S1 Table. Nucleotide sequences of primers and probes used for real time-quantitative PCR

Gene	Primer/probe
α-ttp	5'-GCCAAGAAGATTGCTGCTGTACT-3'F
	5'-ATTTATCAAATGGATCCCACGAA-3'R
	5'-ACAGATTCCTTTCCACTGAA-MGB-3'
β-actin	5'-GCTCTGGCTCCTAGCACCAT-3'F
	5'-GCCACCGATCCACACAGAGT-3'R
	5'-FAM-ATCAAGATCATTGCTCCTC-MGB-3'
18S rRNA	5'-CGGCTACCACATCCAAGGAA-3'F
	5'-GCTGGAATTACCGCGGCT-3'R
	5'-FAM-TGCTGGCACCAGACTTGCCCTC-MGB-3'
GAPDH	5'-CGTCGCGAAGGATACTCT-3'F
	5'-GGCAGCAGATTTCACTGTGAAG-3'R
	5'-FAM-TCGTCAACGGCCACCG-MGB-3'
P. y. 1Cys-Prx	5'-ATCCAGCAACAGGTAGAAATG-3'F
	5'-TCATCATCCTGAAGAGTTGGAATG-R
P. y. Tpx-1	5'-TGCCATCAATTGTAGGAAATCAA-3'F
	5'-CCAAAGGTATTATCACCGAAAACA-3'F
P. y. Hsp-70	5'-TGCTGATAACCAACCAGGAGTCTT-3'
	5'-GCAGGTGGGATACCATCTAAATG-3'
P. y. 18SrRNA	5'-TGAACGAGGAATGCCTAGTAAGC-3'
	5'-TTCATCATATCTTTCAATCGGTAGGA-3'
